# The Feeding of Infants and Children. I.—Health

**Published:** 1892-11-12

**Authors:** 


### THE HOSPITAL CLINIC.

[The Editor will be glad to receive offers of co-operation and contributions from members of the profession. All letters should be addressed to THE EDITOR, THE LODGE, PORCHESTER SQUARE, LONDON, W.]

### PADDINGTON GREEN CHILDREN'S HOSPITAL.

### THE FEEDING OF INFANTS AND CHILDREN.

### I.—IN HEALTH.

The following notes are based on the practice seen in the indoor and outdoor departments of the hospital. In the latter, printed instructions as to diet are supplied to the mothers, with the paragraphs bearing on the individual cases specially marked.

During the first six or seven months breast feeding is always advised where possible, and the printed rule is to "suckle every two hours by day, and twice by night, until the child is three months old ; then suckle every three hours by day, and once only by night." The usual error on the part of mothers is the too frequent suckling, every hour or half-hour, or whenever the baby cries. This soon induces a certain amount of gastric discomfort in the child, to relieve which he will voraciously take the breast whenever opportunity offers. The mother is astonished at his enormous appetite, and brings him to the hospital, saying that she does not think her milk is satisfying, and wishing to know what other food is to be given. A little enquiry elicits the fact that there are also present some sickness after feeding, colic, flatulence, and a tendency to diarrhœa or constipation. A return to the regular hours of feeding, as given above, will soon remedy these evils. The child must not be allowed to gulp down the milk too rapidly, or sickness will usually follow. In some cases, and especially in hot weather, the infant may suffer from thirst, and a few sips of plain water, or barley water, may be given between meals to relieve this condition. Attention is also directed to the nursing mother's diet, and she is advised to drink plenty of cow's milk, and to eat plain, nourishing foods. The routine taking of stout, beer, or spirits is discouraged, as these often produce gastric disturbances in the child. The return of menstruation is not regarded as an indication to stop nursing, provided the health of the mother is good, but nursing is at once stopped should she become pregnant. In those cases in which the mother does not seem to have enough of milk she is advised to continue suckling, and in addition to give the infant some cow's milk and barley water two or three times a day. The administration of any artificial food, bread, or biscuit during the period of lactation is strictly forbidden, except under special medical advice. Weaning is commenced at the end of the seventh month, and cow's milk with some barley water, and later plain cow's milk is given by the bottle or spoon. It is frequently stated by mothers that weaning is impossible, as the child won't take anything except the breast, but practical experience in the wards is entirely opposed to this. In those cases in which breast feeding is abruptly stopped by the admission of the infant into hospital, and bottle feeding is substituted, it is found that, provided the child's alimentary system is in a healthy condition, the change is easily made. A small quantity of fresh cow's milk is mixed with warm barley water and sweetened ; this is offered in the bottle, and if the infant declines it, it is simply put aside and a fresh supply offered when some symptoms of hunger have been manifested. The allowance of pure milk in the twenty-four hours for a healthy infant of three months is placed at a pint and a quarter as an average, but the child's appetite is found to be a better test than any amount fixed by rule, care being taken that the digestion is not overtaxed. The amount of barley water added to the milk varies with the age of the infant ; at one month, two parts of barley water to one of milk, at three months, equal parts, and at six months, one part of barley water to two of milk. The milk is always boiled on delivery at the hospital. The barley water is prepared as follows: Two ounces of barley are well washed in water and then put in a pint and a half of cold water. This is allowed to simmer in a pan for one hour, and then strained through a piece of fine muslin. Boat-shaped feeding bottles, with small rubber teat attached, are the only ones employed. After use they are thoroughly cleansed with hot water and soda, and then placed in a basin of cold water until required. They are not popular amongst mothers, as they cannot be left with the infant like the long tube bottles, but require to be held all the time. This is really an advantage, as it avoids the risk of unobserved choking, and as the rate of absorption can often be regulated better by the mother than by the child. The infant is not allowed to go to sleep with the nipple in its mouth, and the bottle is removed as soon as the appetite is satisfied. The question as to whether young infants should always be wakened at the regular hour for feeding is a somewhat difficult one, but practically it is found that the habit once established is most beneficial; the child is usually ready for food, and will promptly fall asleep again when the meal is over.

After seven months a more varied diet is allowed, but it is strongly impressed on the mothers that up to the age of two years milk must be regarded as the most nourishing food for their children. Boiled bread and milk, oatmeal porridge, gruel, plain puddings made with milk, eggs, potato and gravy, pounded fish, cocoa and milk, stewed fruit, and the juice of fresh ripe fruits form a sufficient variety for a child from nine months up to two years of age. It is advised that the child have at least one pint of cow's milk in the twenty-four hours, and at first one solid meal per diem from the above list may be given, gradually increased to two, and later to three. The system of having young children present at table with their parents is discountenanced, as it almost invariably leads to the child's getting scraps of most unsuitable food. The intervals of feeding are lengthened until the child has four regular meals during the day, and none at night, and no solid food between meals is allowed. The cheapness of artificial foods, and the ease with which they are prepared, have produced a great demand for them ; but mothers are advised to adhere to the natural foods mentioned above, and to let the children have them freshly prepared. The giving of sweets, pastry, cheese, salt fish, tea, wine, beer, or spirits is absolutely forbidden.

In the case of children over two years of age, the home treatment is to let them have the same food as their parents, and it is practically impossible for the mothers to have a special dietary for them. They are advised, however, to limit the amount of tea and butcher's meat given to the children. The following is the hospital programme of meals for children who are over two years of age and who are on full diet:—

Breakfast at 6 a.m.—Bread and dripping, hot milk.

At 10 a.m.—Milk alone.

Dinner at noon.—Fish or meat with potatoes, milk puddings, milk to drink, bread.

Tea at 4 p.m.—Cocoa and milk, bread and dripping ; eggs every alternate day.

At 6.30 p.m.—Milk alone.

